# Associations of fitness, motor competence, and adiposity with the indicators of physical activity intensity during different physical activities in children

**DOI:** 10.1038/s41598-021-92040-2

**Published:** 2021-06-15

**Authors:** Eero A. Haapala, Ying Gao, Jani Hartikainen, Timo Rantalainen, Taija Finni

**Affiliations:** 1 Faculty of Sport and Health Sciences, University of Jyväskylä, PO Box 35, 40014 Jyväskylä, Finland; 2 Institute of Biomedicine, School of Medicine, University of Eastern Finland, Kuopio, Finland; 3 Department of Sports Science, College of Education, Zhejiang University, Hangzhou, China

**Keywords:** Physiology, Epidemiology

## Abstract

We investigated the associations of peak oxygen uptake (V̇O_2peak_), ventilatory threshold (VT), muscle strength, motor competence (MC), and adiposity with the indicators of PA intensity during different physical activities used to create absolute PA intensity cut-offs among 35 children 7–11-years-of-age. V̇O_2peak_ was defined as the highest V̇O_2_ achieved in the maximal cardiopulmonary exercise test (CPET) on a cycle ergometer, self-paced running, or running on a treadmill at 8 km/h. VT was defined from the CPET data. Peak isometric knee extensor and flexor strength was assessed by a dynamometer, MC by the Körperkoordination test für Kinder tests, and body composition by the bioelectrical impedance analysis. PA intensity was assessed using V̇O_2_ as a % of V̇O_2reserve_ or V̇O_2_ at VT, mean amplitude deviation (MAD) measured by accelerometry, metabolic equivalent of task (MET), and muscle activity measured by textile electromyography during walking or running on a treadmill at 4, 6, and 8 km/h, playing hopscotch, walking up and down the stairs, self-paced walking, and self-paced running. Children with lower V̇O_2peak_, V̇O_2_ at VT, and MC operated at higher intensity level during given physical task than their peers with higher fitness and MC when PA intensity was expressed using relative PA intensity using V̇O_2_ as a % of V̇O_2reserve_ or V̇O_2_ at VT (*p* < 0.05). MAD and METs during different tasks were not able to discriminate PA intensity between children with varying levels of physical fitness or MC. Traditionally used absolute measures of PA intensity based on accelerometry or MET underestimated PA intensity in children with lower V̇O_2peak_, V̇O_2_ at VT, and MC.

## Introduction

Physical inactivity in childhood has been considered one of the most important public health issues globally^1^. Physical activity (PA) has been positively associated with cardiorespiratory fitness (CRF), motor competence (MC), and muscle strength in children and some evidence indicates that vigorous PA has the strongest associations with these outcomes^2–4^. PA has also been inversely associated with body fat percentage (BF%) in children^3^. However, to the best of our knowledge, there are no previous studies on the associations of CRF, MC, muscle strength, and adiposity with the indicators of PA intensity in different laboratory tasks, which have been used to create PA intensity cut-offs for the assessment of habitual PA among children^5,6^. Such information is essential to provide the best evidence to inform PA recommendations and to understand the role of PA in health and wellbeing in children.

Peak oxygen uptake (V̇O_2peak_) refers to the maximal capacity of cardiopulmonary and vascular systems to deliver oxygen to working skeletal muscles and the ability of skeletal muscles to extract oxygen from the blood and use it in aerobic energy metabolism^7^. Therefore, V̇O_2peak_ stands for the highest intensity where energy metabolism can be supported by aerobic energy production and therefore it has been considered the gold standard in the assessment of CRF^7^. However, because children rarely perform activities near their V̇O_2peak_ during their daily lives^8^, V̇O_2_ at ventilatory threshold (VT) has been considered an important submaximal measure of CRF^9^. In exercise physiology, V̇O_2_ at VT has been considered the physiological threshold for vigorous intensity PA (VPA)^10^ reflecting the metabolic changes in the skeletal muscle due to elevated lactate concentration during increasing exercise intensity^9^.

V̇O_2peak_ and V̇O_2_ at VT reflects cardiovascular and metabolic capacity to perform daily activities, but several common activities performed by children require sufficient levels of neuromuscular control and capacity, which can be quantified as MC and muscle strength^11,12^. MC can be defined as the competent and purposeful movement and the ability to flexibly adapt the movement in changing environments^11^ while muscle strength can be broadly defined as the ability to produce force against an external load^12^. Although MC and muscle strength are sometimes considered separate entities, competent performance of various motor tasks requires sufficient levels of muscle strength^13^.

In observational studies PA intensity is often expressed using absolute cut-offs based on metabolic equivalent of tasks (METs) or acceleration magnitude derived from accelerometers^14^ and most studies on the associations of PA with CRF, MC, muscle strength, and adiposity have utilised these absolute PA intensity cut-offs^2–4^. However, these absolute PA intensity cut-offs assess the ability of an individual to reach a certain level of energy expenditure or acceleration without taking into account large interindividual variation in cardiovascular, metabolic, and neuromuscular capacity^14^. Furthermore, the absolute cut-offs used in paediatric research has been developed using pre-determined calibration activities, e.g. defining that walking on a treadmill for 6 km/h and walking up and down the stairs reflects moderate intensity PA, without taking individual exercise capacity in to account^5,6^. Therefore, V̇O_2_ as a proportion of V̇O_2peak_ (i.e. V̇O_2reserve_), V̇O_2_ as a % of V̇O_2_ at VT, and muscle activity measured by electromyography (EMG) have been considered recommended methods to express relative PA intensity because they take into account individual variation in exercise capacity^14–16^.

Previous findings in adults suggest that reaching absolute PA intensity cut-offs may be harder for unfit and overweight and obese adults than for higher fit and normal weight adults^17,18^. However, relative PA intensity cut-offs set individually using either V̇O_2reserve_ or V̇O_2_ at VT reduce the differences in PA volume between adults with varying levels of V̇O_2peak_ and body mass index^17,18^. While evaluating PA intensity using V̇O_2reserve_ instead of absolute PA intensity cut-offs is preferred, anchoring PA intensity to VT may provide physiologically more appropriate estimate of PA intensity because VT takes individual physiological responses to exercise into account better than V̇O_2reserve_^19^.

Children with higher V̇O_2peak_, MC, muscle strength, and lower adiposity have been found to accumulate more moderate-to-vigorous PA than other children in observational studies^6,8,20^. However, there are no previous studies investigating whether V̇O_2peak_, V̇O_2_ at VT, MC, muscle strength, and adiposity are associated with indicators of PA intensity based on V̇O_2reserve_ or V̇O_2_ at VT during different activities commonly used in accelerometry cut-off validation studies^5,21^. Furthermore, we are not aware of previous studies in children investigating whether PA intensity assessed by accelerometry or METs during those activities can capture differences in physical fitness or adiposity. We have previously observed that V̇O_2_ as a % of V̇O_2reserve_ and V̇O_2_ at VT varies from 30 to 100% of V̇O_2reserve_ and 50–180% of V̇O_2_ at VT running on a treadmill for 6 and 8 km/h, PA intensities considered moderate and vigorous in previous studies, suggesting remarkable interindividual variation in physiological responses during these absolute treadmill speeds^22^. Therefore, to better understand and interpret the results of observations studies on the associations of the measures of physical fitness, MC, and adiposity and other health outcomes with PA it is essential to investigate the role of physical fitness, MC, and adiposity in different activities utilised to validate commonly used PA intensity cut-offs.

Our first aim was to investigate the associations of V̇O_2peak_/kg of skeletal muscle mass (SMM), V̇O_2_ at VT/SMM, MC, muscle strength, and BF% with relative PA intensity quantified as V̇O_2reserve_ and VT during different activities. The second aim was to study whether the associations of V̇O_2peak_/kg of SMM, V̇O_2_ at VT/SMM, MC, muscle strength, and BF% with mean amplitude deviations (MAD) and METs, which are absolute measures of PA intensity agree with the those of relative PA intensity indicators. For completeness, we also report the associations of V̇O_2peak_/kg of SMM, V̇O_2_ at VT/SMM, MC, muscle strength, and BF% with muscle activity during different activities measured using EMG.

## Methods

This study was based on the laboratory phase of the Children’s Physical Activity Spectrum (CHIPASE) study^23^. A total of 35 children (21 girls; 14 boys) aged 7–11 years were recruited from local schools and volunteered to participate in the study. Children were included if they were apparently healthy and were able to perform the physical activities at moderate and vigorous intensities. Children with chronic conditions or disabilities were excluded from the study. The study protocol was approved by the Ethics Committee of the University of Jyväskylä (28.8.2017). All children gave their assents and their parents/caregivers gave their written informed consents. The study was conducted in agreement with the Declaration of Helsinki.

Based on the main research question of the CHIPASE Study, a sample size of 30 was estimated to provide sufficient statistical power for differentiating METs between sitting (1.33 ± 0.24) and standing (1.59 ± 0.37) based on the data of Mansoubi et al^24^ with 80% power and 5% α-error level.

### Study protocol

The participants visited laboratory three times as described previously^22^. At the first visit, research staff explained the research protocol to children and their parents. They were also familiarised to the laboratory environment and measurement equipment. At the second visit, children arrived at the laboratory in the morning after 10–12 h overnight fast for assessment of anthropometrics, body composition, resting V̇O_2_, and MC and muscle strength. Children consumed a breakfast after the assessment of resting V̇O_2_ before the assessment of MC and muscle strength. At the third visit, children were asked to perform following activities for 4.5 min in a random order interspersed with 1-min rest: sitting quietly, sitting while playing a mobile game, standing quietly, standing while playing a mobile game, playing hopscotch, walking up and down the stairs, and walking or running on a treadmill at 4, 6, and 8 km/h. They were also asked to walk and run around an indoor track at self-chosen speed for 4.5 min. For the self-paced tasks, children were instructed to perform the tasks at their own comfortable pace without rushing or slowing down (e.g. walk at the same speed as you would walk to school; run at a speed that fits for you and you can run a little longer distance). At the end of the third visit, children performed maximal cardiopulmonary exercise test on a bicycle ergometer. During the third visit, children did not consume snack or beverages during the course of the assessment but they were allowed to drink water at will. During the activities, we confirmed that V̇O_2_ returned near to baseline levels during the 1-min rest^22^. Children also rested approximately 30 min before the maximal bicycle ergometer test after they had completed other activities. Concurrent recording of V̇O_2_ by portable respiratory gas analyser, movement by triaxial accelerometer, and muscle activity by the textile EMG shorts were conducted during the activities. The activities used in this study have been used in previous calibration studies^5,21,25^ and they also mimic normal daily activities performed by children.

### Assessments

#### Body size and body composition

Stature was measured to the nearest 0.1 cm using a wall-mounted stadiometer^22^. Body mass (BM), SMM, fat mass, fat free mass, and body fat percent were measured by InBody 770 bioelectrical impedance device (Biospace Ltd., Seoul, Korea). A relatively good agreement between SMM assessed by bioelectrical impedance and lean mass assessed by dual-energy X-ray absorptiometry have been reported in children^26^. Furthermore, we observed a strong correlation between SMM and fat free mass in the present study (*r* = 0.997, *p* < 0.001). Body mass index (BMI) was calculated by dividing body weight with body height squared and body mass index standard deviation score (BMI-SDS) was computed using the Finnish references^27^.

#### Peak oxygen uptake and oxygen uptake at ventilatory threshold

Cardiorespiratory fitness was assessed by a maximal ramp exercise test on an electromagnetically braked Ergoselect 200 K® electromagnetic cycle ergometer (Ergoline, Bitz, Germany) as described previously^22,28^. Shortly, the protocol included 2-min resting period sitting on an ergometer, a 3-min warm-up with a workload of 20 W, and an incremental exercise period with increase of workload either by 1 W/3 s (totalling 20 W/min for children > 150 cm), 1 W/4 s (totalling 15 W/min for children 126–150 cm), or 1 W/6 s (totalling 10 W/min for children ≤ 125 cm) until voluntary exhaustion^29^. The participants were asked to maintain the cadence at 70–80 during the test. The test was terminated when the participant was unable to keep the cadence of 65 or required to stop. Participants were verbally encouraged to exercise until voluntary exhaustion.

Respiratory gas exchange was assessed directly by breadth-by-breadth method using the metabolic cart from the 2-min resting period sitting on the ergometer until the voluntary exhaustion and were averaged over 15-s periods. We defined peak cardiorespiratory capacity as the highest V̇O_2_ achieved in the exercise test (V̇O_2peak_) averaged over 15 s recorded during the last minute of the exercise test and normalised it for SMM. We normalised V̇O_2peak_ for SMM because it has been suggested that normalising CRF for SMM provides physiologically the most appropriate measure of peak aerobic capacity independent of body size and body composition^30^. The results remained unchanged when we used fat free mass as a scaling factor. If higher V̇O_2_ was observed during running on a treadmill for 8 km/h or during self-paced running (N = 21) than during maximal cycle exercise tests (N = 14), the higher V̇O_2_ value was used as a measure of V̇O_2peak_. Beat-by-beat heart rate (HR) was continuously recorded during the exercise test using Polar H7 HR sensor (Polar Electro, Kempele, Finland).

The cardiopulmonary exercise test was considered maximal if the primary and secondary objective and subjective criteria indicated maximal effort and maximal cardiorespiratory capacity (a plateau of V̇O_2_ regardless of increasing workload, HR > 85% of predicted, respiratory exchange ratio > 1.00, or flushing and sweating), and the exercise physiologist supervising the exercise test considered the test maximal^7^.

V̇O_2_ at VT was determined individually by two exercise physiologists using modified V-slope method^31^ and any disagreements were solved by these two exercise physiologists^22^. The VT was identified as a time point the increase in V̇CO_2_ is steeper than the increase in V̇O_2_ during the maximal cardiopulmonary exercise test on a cycle ergometer. In determination of VT, we used data averaged over 15 seconds^31^ normalised for SMM. V̇O_2_ at VT was verified utilising the equivalents for V̇_E_/V̇CO_2_ and V̇_E_/V̇O_2_. According to equivalent method V̇O_2_ at VT was defined as a rate of V̇O_2_ where V̇_E_/V̇O_2_ begins to increase without an increase in V̇_E_/V̇CO_2_.

#### Motor competence and muscle strength

A proxy for MC was assessed by the Körperkoordination test für Kinder^32^. During the assessment, children were asked to (1) walk backwards on balance beams with decreasing widths of 6.0 cm, 4.5 cm, and 3.0 cm, (2) hop for height on one foot at a time, over a pile of soft mattresses (width 60 cm; depth 20 cm; height 5 cm each) with increasing height after each successful attempt, (3) jump sideways from side to side over a thin wooden lath (60 × 4 × 2 cm) on the jumping base (100 × 60 cm), and (4) move sideways with wooden plates (size 25 × 25 cm; height 5.7 cm) without stepping out as quickly as possible for 20 s. We calculated the MC score from the sample specific z-scores by summing-up these four z-scores. A higher MC indicates better MC.

Peak isometric knee extensor and flexor strength was assessed using knee extensor and knee flexion dynamometers (David ltd, Helsinki, Finland). After three submaximal trials, children were asked to maximally extend or flex their knee during the task. Children had three maximal trials. Muscle strength was defined as a mean of the maximal extension and flexion force in Newtons normalised using log-linear allometry for kg of SMM^1.77^.

### Oxygen uptake, accelerometry, and electromyography during different physical activities

#### Oxygen uptake

Mobile metabolic cart (Oxycon mobile, CareFusion Corp, USA) was calibrated and dead space was adjusted to 78 ml for the petite size of the face mask following the manufacturer’s recommendations^22,23^. V̇O_2_, carbon dioxide production (V̇CO_2_) and respiratory exchange ratio were collected breath by breath and computed in non-overlapping 1 s epoch lengths. Resting V̇O_2_ was determined as the mean value between the 15th and 25th minute of 30 min of supine rest when the steady state was reached^33^. When steady stated was not observed between 15 and 25th minute, the steady state was visually selected for further analysis. In physical activities, V̇O_2_ was averaged over 2 min from the 3rd and 4th minutes of each task when plateau in V̇O_2_ and V̇CO_2_ was observed^23,34^. V̇O_2_ reserve as a percentage of V̇O_2peak_ during different physical activities was calculated as (V̇O_2_ during PA task/V̇O_2peak_—V̇O_2_ during rest) × 100. Metabolic equivalent of task (MET) values were computed as V̇O_2_ measured during the physical activities/V̇O_2_ during supine rest.

#### Accelerometry

Movement was measured by triaxial accelerometer (X6-1a, Gulf Coast Data Concepts Inc., Waveland, USA)^23^. We used raw acceleration data in actual g-units with the high range up to 6 g with 16-bit A/D conversion and sampling at 40 Hz. The resultant acceleration of the triaxial accelerometer signal was calculated from $\sqrt {x^{2} + y^{2} + z^{2} }$, where x, y and z are the measurement sample of the raw acceleration signal in x-, y-, and z-directions. The X6-1a accelerometer has been shown to produce congruent results with the ActiGraph GT3X accelerometer^35^. The mean amplitude deviation (MAD) was calculated from the resultant acceleration in non-overlapping 1 s epoch. MAD described as the mean distance of data points about the mean ($\frac{1}{n}\mathop \sum \limits_{{i = 1}}^{n} \left| {r_{{i~ - }} \bar{r}} \right|$ where n is the number of samples in the epoch, $r_{{i~}}$ is the *i*th resultant sample within the epoch and $\bar{r}$ is the mean resultant value of the epoch)^25,35^. The mean of the 1 s MAD values (g) were calculated in the 2 min time epochs for each activity and in 10-min epoch for lying down and are reported as the outcomes corresponding to the steady state intervals of the V̇O_2_ measurements. MAD values derived from the raw acceleration signal have been found to be independent of accelerometer brand and therefore provide an universal method to assess PA intensity across studies and triaxial accelerometry devices^25^.

#### Textile electromyography

Textile EMG electrodes embedded into elastic garments were used to assess muscle activity from the quadriceps and the hamstring muscles and has been described in detail previously^23^. We have previously showed that day-to-day coefficient variation ranged from 4 to 11% suggesting a good repeatability of textile EMG with a high agreement of textile EMG with a traditional surface EMG^36^. Four different sizes of EMG shorts (120, 130, 140, and 150 cm) with zippers located at the inner sides of short legs and adhesive elastic band in the hem ensured proper fit in every child. The conductive area of the electrodes over the muscle bellies of the left and the right quadriceps was 9 × 2 cm^2^ (length × width) in all short sizes, while the corresponding sizes for the hamstring muscles were 6 × 2 cm^2^ in sizes of 120, 130, and 140 cm and 6.5 × 2 cm^2^ in size of 150 cm. The conductive area of the reference electrodes was 11 × 2 cm^2^, and they were located longitudinally over the iliotibial band. Water or electrode gel (Parker Laboratories Inc., Fairfield, NJ, USA) was used on the electrode surfaces to minimize the skin–electrode impedance.

In the signal analysis, EMG data were identified from different activities in the certain time windows simultaneously according to the steady state in respiratory gases. Individual EMG activities were normalised channel by channel to EMG amplitude measured during self-paced walking. The normalised EMG data were averaged for quadriceps from right and left side and hamstring muscles from right and left side, then the mean amplitude of the average normalised data was computed as the intensity of muscle activity level for each activity.

### Statistical methods

Basic characteristics between girls and boys were compared using Student’s t-test for normally distributed continuous variables and Mann–Whitney U-test for skewed continuous variables. We investigated the correlations between V̇O_2peak_, V̇O_2_ at VT, MC, peak isometric strength, and adiposity and V̇O_2_ as a % of V̇O_2_, V̇O_2_ as a % of V̇O_2_ at VT, MAD, and EMG reserve during different activities using Spearman correlation coefficients. Differences in V̇O_2_ as a % of V̇O_2reserve_, V̇O2 as a % of V̇O2 at VT, MAD, and EMG among children divided to three equal size groups (thirds) of V̇O_2peak_ normalised for SMM, V̇O_2_ at VT normalised for SMM, MC, peak isometric strength normalised for SMM^1.77^, and adiposity were investigated using Kruskal–Wallis test. Student´s t-test, the Mann–Whitney U-test, and the χ2 test were performed using the SPSS Statistics, Version 23.0 (IBM Corp., Armonk, NY, USA). The data were visualised and Spearman correlations and Kruskal–Wallis tests were performed by the GraphPad Prism, version 8.0.2 (Graph Pad Software, Inc., San Diego, CA, USA). Because of some missing data due to the poor data quality or device malfunction, the sample size in different tasks varied from 29 to 35 participants. Analyses were performed using the maximum number of participants with valid data. Because of the large number of statistical analyses, we utilised Bonferroni correction for multiple testing in the correlation analyses. Correction for multiple testing was performed for each activity intensity indicator. Bonferroni-corrected *p*-values were computed by dividing the *p*-value of 0.05 by the number of variables used in each analysis resulting in the corrected critical value of 0.01.

### Ethics approval

The study protocol was approved by the Ethics Committee of the University of Jyväskylä.

### Consent to participate

All children gave their assents and their parents/caregivers gave their written informed consents. The study was conducted in agreement with the Declaration of Helsinki.

## Results

### Basic characteristics and correlations between V̇O2peak, VT, motor competence, peak isometric strength, and adiposity

Girls were lighter, had lower BMI, and had less fat mass and SMM than boys (Table 1). Girls also had lower resting V̇O_2_ and higher peak isometric strength than boys (Table 1). V̇O_2peak_ correlated positively to V̇O_2_ at VT. MC correlated negatively to BF% and positively to peak isometric strength (Table 2).

**Table 1:** Characteristics of participants.

	All	Girls	Boys	*P*-value
Age (years)^a^	9.6 (3.0)	9.7 (2.9)	9.6 (2.8)	0.960
Stature (cm)	137.6 (9.2)	135.7 (9.3)	140.4 (8.7)	0.149
Weight (kg)	32.6 (6.9)	30.2 (6.0)	36.2 (6.8)	**0.009**
BMI (kg/m^2^)^a^	16.5 (3.6)	16.1 (2.2)	17.7 (4.8)	**0.037**
BMI-standard deviation score	− 0.2 (1.2)	− 0.5 (1.1)	0.3 (1.2)	0.052
Fat mass (kg)^a^	4.8 (5.0)	4.2 (4.2)	6.8 (7.5)	**0.022**
Body fat percentage (%)	16.6 (8.1)	15.7 (7.3)	18.0 (9.3)	0.433
Skeletal muscle mass (kg)	14.0 (2.9)	13.0 (2.5)	15.5 (2.8)	**0.009**
Resting V̇O_2_ (mL/min)^a^	153.9 (32.8)	146.4 (26.2)	189.3 (63.9)	**0.006**
V̇O_2peak_ (mL/kg of SMM^−1^/min)^a^	103 (17.2)	98.4 (19.8)	103.8 (9.4)	0.576
V̇O_2peak_ (mL/kg of BM^−1^/min)^a^	44.9 (9.6)	43.8 (11.1)	46.3 (10.2)	0.753
V̇O_2_ at VT (mL/kg of SMM^−1^/min^−1^) on a maximal cycle ergometer exercise test	67.2 (9.8)	69.1 (11.7)	65.8 (5.6)	0.287
Motor competence^b^	0.07 (3.5)	0.38 (3.4)	− 0.37 (3.6)	0.567
Peak isometric knee extensor and flexor strength (Newtons)	78.8 (26.5)	76.5 (25.5)	82.4 (28.8)	0.554
Peak isometric knee extensor and flexor strength (Newtons, sum score)/SMM^1.77^	0.7 (0.14)	0.8 (0.1)	0.6 (0.1)	**0.009**
Peak isometric knee extensor and flexor strength (Newtons, sum score)/kg of body mass	2.4 (0.6)	2.4 (0.6)	2.3 (0.7)	0.586

The data are mean (SD) or ^a^median (IQR). V̇O_2peak_ was defined as the highest V̇O_2peak_ during running on a treadmill for 8 km/h, self-paced running, or on a maximal cycle ergometer exercise test; V̇O_2_ = oxygen uptake; SMM = skeletal muscle mass. *P* values refer to statistical significance for differences between girls and boys with statistically significant differences bolded. ^b^Motor competence score was computed using the sample specific KTK result z-scores.

**Table 2:** Spearman correlation coefficients between the measures of cardiorespiratory fitness, motor competence, muscle strength, and adiposity.

	V̇O_2peak_/kg of SMM/min	V̇O_2_ at VT/kg of SMM	Motor competence	Peak isometric strength (N)/SMM^1.77^
V̇O_2_ at VT/kg of SMM	0.370*	–		
Motor competence	0.030	0.140	–	
Peak isometric strength (N)/SMM^1.77^	0.340	0.165	0.386*	–
Body fat percentage	− 0.136	0.202	− 0.424*	− 0.040

V̇O_2peak_/kg of SMM/min = peak oxygen uptake scaled by skeletal muscle mass (SMM); V̇O_2_ at VT/kg of SMM = oxygen uptake at ventilatory threshold scaled by skeletal muscle mass; N = Newtons. *p < 0.05. Statistically significant associations are given in bold.

### Correlations of V̇O_2peak_, VT, motor competence, peak isometric strength, and adiposity with the indicators of physical activity intensity in different tasks

#### Peak oxygen uptake

V̇O_2peak_ correlated negatively with V̇O_2_ as % of V̇O_2reserve_ during walking or running on treadmill for 6 and 8 km/h, climbing up and down the stairs and during playing hopscotch (Table 3). Higher V̇O_2peak_ was associated with higher MAD during self-paced walking and METs during self-paced running. The effect of correction for multiple testing using the Bonferroni correction is demonstrated in Table 3.

**Table 3:** Spearman correlations coefficients of cardiorespiratory fitness, motor competence, muscle strength, and adiposity to physical activity intensity in different tasks.

	Treadmill			Climbing up and down the stairs	Playing hopscotch	Indoor track	
	4 km/h	6 km/h	8 km/h			Self-paced walking	Self-paced running
**Intensity relative VO**_**2reserve**_							
V̇O_2peak_/kg of SMM/min	− 0.339	− 0.480**^a^	− 0.562**^a^	− 0.517**^a^	− 0.655***^a^	− 0.274	0.179
V̇O_2_ at VT/kg of SMM	− 0.121	− 0.122	− 0.093	− 0.508**^a^	− 0.203	− 0.125	− 0.186
Motor competence	− 0.165	− 0.269	− 0.238	− 0.313	− 0.088	0.060	− 0.052
Peak isometric strength (N)/SMM^1.77^	0.009	− 0.097	− 0.167	− 0.317	− 0.040	− 0.234	− 0.234
Body fat percentage	0.228	0.240	0.349	0.489**^a^	0.211	− 0.223	0.106
**Intensity relative to ventilatory threshold (V̇O**_**2**_** as % of V̇O**_**2**_** at ventilator threshold)**							
V̇O_2peak_/kg of SMM/min	− 0.140	− 0.231	− 0.157	− 0.325	− 0.307	− 0.003	0.247
V̇O_2_ at VT/kg of SMM	− 0.508**^a^	− 0.482**^a^	− 0.590**^a^	− 0.699***^a^	− 0.592**^a^	− 0.536**^a^	− 0.688***^a^
Motor competence	− 0.408*	− 0.572**^a^	− 0.567**^a^	− 0.406*	− 0.341	− 0.134	− 0.356
Peak isometric strength (N)/SMM^1.77^	− 0.099	− 0.188	− 0.157	− 0.351	− 0.070	0.029	− 0.075
Body fat percentage	0.003	0.050	0.149	0.358*	0.124	− 0.219	− 0.024
**Intensity assessed by mean amplitude deviation**							
V̇O_2peak_/kg of SMM/min	0.157	− 0.031	0.115	− 0.004	0.078	0.500**^a^	0.282
V̇O_2_ at VT/kg of SMM	− 0.075	− 0.026	0.065	− 0.449*	0.116	0.001	0.004
Motor competence	− 0.700***	− 0.382*	0.577**	− 0.117	0.608**	− 0.010	0.684***
Peak isometric strength (N)/SMM^1.77^	− 0.316	− 0.105	0.208	− 0.281	0.496**	0.235	0.094
Body fat percentage	0.260	0.100	− 0.269	− 0.032	− 0.051	− 0.171	− 0.409*
**Intensity based on metabolic equivalent of tasks**							
V̇O_2peak_/kg of SMM/min	− 0.034	− 0.155	0.036	− 0.239	− 0.203	0.116	0.545**
V̇O_2_ at VT/kg of SMM	0.023	0.158	0.132	− 0.230	0.086	0.067	0.138
Motor competence	− 0.265	0.351	− 0.085	− 0.158	− 0.064	0.068	0.163
Peak isometric strength (N)/SMM^1.77^	0.087	− 0.019	0.225	− 0.171	0.144	0.151	0.311
Body fat percentage	0.166	0.107	0.196	0.350*	0.144	− 0.239	− 0.061
**Intensity relative to EMG normalised for EMG measured during self-paced walking**							
V̇O_2peak_/kg of SMM/min	− 0.127	− 0.010	− 0.168	0.096	0.021	–	0.008
V̇O_2_ at VT/kg of SMM	− 0.203	− 0.231	− 0.331	− 0.376*	− 0.234	–	0.127
Motor competence	− 0.545**	− 0.388*	− 0.258	− 0.025	0.305	–	0.489*
Peak isometric strength (N)/SMM^1.77^	− 0.342	− 0.264	− 0.275	− 0.159	0.070	–	0.159
Body fat percentage	0.461**	0.274	0.284	0.102	0.131	–	− 0.018

V̇O_2peak_/kg of SMM/min = peak oxygen uptake scaled by skeletal muscle mass (SMM); V̇O_2_ at VT/kg of SMM = oxygen uptake at ventilatory threshold scaled by skeletal muscle mass; N = Newtons. *p < 0.05, **p < 0.01, ***p < 0.001. Table includes both non-corrected and corrected significances. Statistically significant non-corrected associations are given in bold. ^a^Statistically significant after the Bonferroni correction.

#### *V̇O*_*2*_* at ventilatory threshold*

V̇O_2_ at VT was inversely associated with V̇O_2_% of V̇O_2reserve_ during climbing up and down the stairs (Table 3). Higher V̇O2 at VT was associated with lower V̇O_2_ as % of V̇O_2_ at VT during all PAs and lower MAD and EMG during climbing up and down the stairs. The effect of correction for multiple testing using the Bonferroni correction is demonstrated in Table 3.

#### Motor competence

MC was inversely associated with V̇O_2_ as % of V̇O_2_ at VT during walking or running on treadmill for 4, 6 and 8 km/h, and climbing up and down the stairs (Table 3). Better MC was associated with lower MAD during walking or running on treadmill for 4 and 6 km/h and with higher MAD during running on a treadmill for 8 km/h, playing hopscotch and self-paced running. MC was also inversely associated with EMG during walking or running on treadmill for 4 and 6 km/h and positively associated with EMG during self-paced running (Table 3). The effect of correction for multiple testing using the Bonferroni correction is demonstrated in Table 3.

#### Muscle strength

Peak isometric strength was positively associated with MAD during playing hopscotch (Table 3). Peak isometric strength was not associated with any other indicator of PA intensity. The effect of correction for multiple testing using the Bonferroni correction is demonstrated in Table 3.

#### Body fat percentage

BF% was positively associated with V̇O_2_% of V̇O_2reserve_ and V̇O_2_ as % of V̇O_2_ at VT during climbing up and down the stairs (Table 3). BF% was inversely associated with MAD during self-paced running. BF% was also positively correlated to METs during climbing up and down the stairs and to EMG during walking treadmill for 4 km/h. The effect of correction for multiple testing using the Bonferroni correction is demonstrated in Table 3.

### Differences in physical activity intensity between thirds

**Figure 1:**
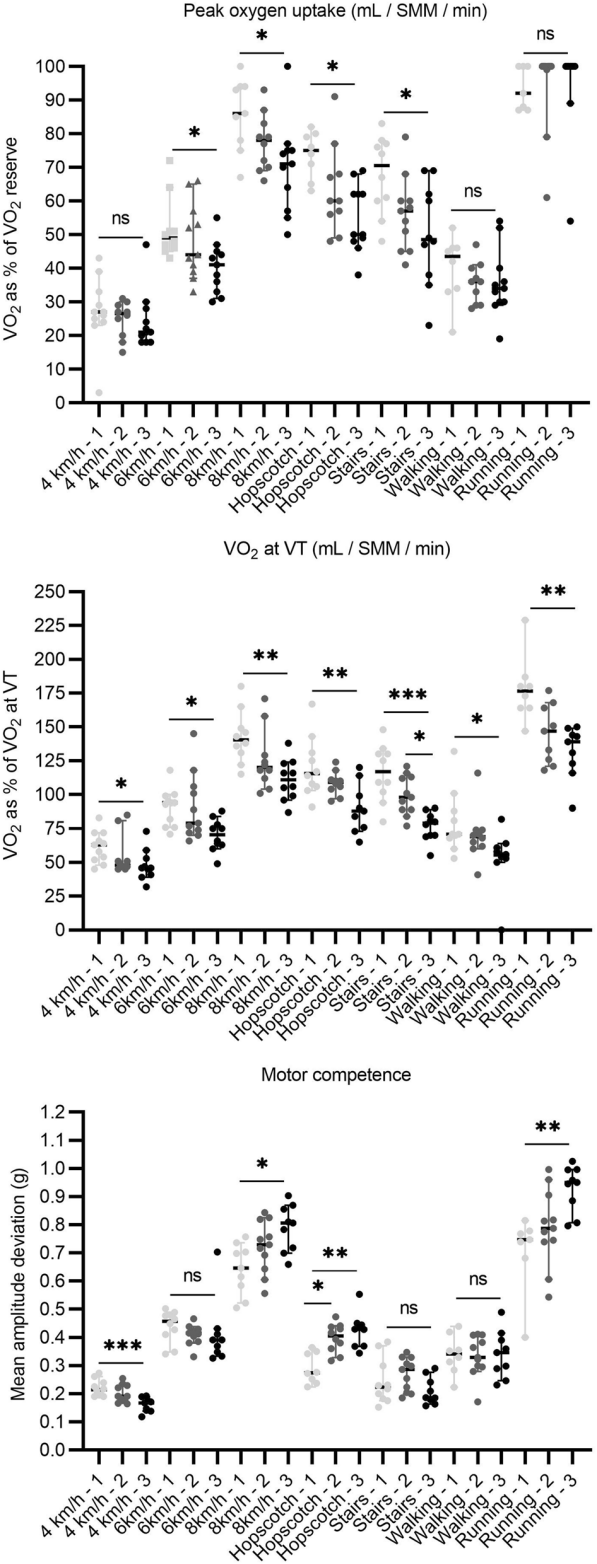
Differences in the different measures of physical activity intensity indices among the thirds of measures physical fitness and motor competence. *p < 0.05, **p < 0.01, ***p < 0.001, ns = statistically insignificant. VO2 = oxygen uptake, VT = ventilatory threshold.

Children in the lowest third of V̇O_2peak_ had higher V̇O_2_ as % of V̇O_2reserve_ during walking or running on a treadmill for 6 km/h (*p* = 0.024 for the main effect) and 8 km/h (*p* = 0.020 for the main effect), playing hopscotch (*p* = 0.005 for the main effect), and climbing up and down the stairs (*p* = 0.029 for the main effect) than those in the highest third of V̇O_2peak_ (Fig. 1). The main effect for MET during self-paced running was also significant (*p* = 0.036), but post-hoc tests did not reveal statistically significant differences in METs between the thirds of V̇O_2peak_ (*p* = 0.07 for difference).

Children in the lowest third of V̇O_2_ at VT operated at higher level relative to their VT during walking or running on treadmill for 4 km/h (*p* = 0.032 for the main effect), 6 km/h (*p* = 0.012 for the main effect), and 8 km/h (*p* = 0.007 for the main effect), playing hopscotch (*p* = 0.011 for the main effect), walking up and down the stairs (*p* < 0.001 for the main effect), self-paced walking (*p* = 0.013 for the main effect), and self-paced running (*p* = 0.002 for the main effect) than those in the highest third of V̇O_2_ at VT. Furthermore, children in the middle third of VT operated at higher level relative to their VT than those in the highest third during climbing up and down the stairs.

Children in the lowest third of MC operated at higher level relative to their VT than children in the highest third of MC during walking or running on a treadmill for 6 km/h (*p* = 0.039 for the main effect). Moreover, children in the middle third of MC operated at higher level relative to their VT during playing hopscotch than those in the highest third (*p* = 0.022 for the main effect). The main effect of MC for VT was significant (*p* = 0.048 for the main effect) for 8 km/h, but post-hoc tests showed no statistically significant differences between thirds (*p* = 0.054 for difference). Children in the lowest third of MC had higher MAD during walking on a treadmill for 4 km/h (*p* = 0.001 for the main effect) and lower MAD during running on a treadmill for 8 km/h (*p* = 0.015 for the main effect), playing hopscotch (*p* = 0.001 for the main effect), and self-paced running (*p* = 0.005 for the main effect). Children in the lowest third of MC had also higher MAD during playing hopscotch than those in the middle third of MC.

## Discussion

We found that children with lower V̇O_2peak_ and V̇O_2_ at VT operated at higher intensity level during given physical activity than their peers with higher fitness. We also observed that children with better MC operated at lower intensity level relative to their VT. Despite the significant differences in the indicators of relative PA intensity between children with lower and higher V̇O_2peak_, V̇O_2_ at VT, and MC, the associations of V̇O_2peak_, V̇O_2_ at VT, and MC with MAD and METs during different tasks were weak and inconsistent. Therefore, these results suggest that absolute MAD and METs were not able to discriminate physiological strain between children with lower and higher fitness and MC. These results are in line with previous findings in adults showing that higher fit individuals can reach absolute PA intensity cut-offs more easily than lower fit individuals^17,20^.

We found that children with higher V̇O_2peak_ and V̇O_2_ at VT operated at lower level relative to their maximal physiological capacity, than those with lower V̇O_2peak_ and V̇O_2_ at VT during most PA tasks. Nevertheless, we found no statistically significant differences in MAD and METs measured during different PA tasks between children in the thirds of V̇O_2peak_ and V̇O_2_ at VT. Furthermore, previous studies have shown large differences in moderate-to-vigorous PA between lower and higher fit adults when PA intensity was based on absolute cut-offs^17,18^. Nevertheless, those differences reduced remarkably when PA intensity was related to individual exercise capacity^17,18^. Furthermore, most studies providing the absolute acceleration magnitude cut-offs for PA intensity in children have used pre-determined calibration tasks to define PA intensity. In those studies walking on a treadmill for 4 km/h has been considered light PA, running on treadmill for 6 km/h, walking up and down the stairs playing hopscotch, and walking around an indoor track on self-chosen speed as moderate PA, and running on a treadmill for 8 km/h and running around an indoor track on self-chosen speed as vigorous PA^5,6^. Therefore, these results together suggest that absolute acceleration magnitude cut-offs penalise children with lower fitness level and may cause bias on the association of PA with V̇O_2peak_ and V̇O_2_ at VT.

Children with higher V̇O_2peak_ achieved higher MET values during self-paced running although there were no differences in proportion of V̇O_2reserve_ during self-paced running between children in the thirds of V̇O_2peak_. Similarly, V̇O_2peak_ was positively associated with MAD during self-paced walking whereas we observed no differences in % of V̇O_2reserve_ during self-paced walking between children with varying levels of V̇O_2peak_. These results suggest that children with higher V̇O_2peak_ walk and run faster at self-chosen speed, causing higher MAD and MET values. Furthermore, we did not observe other statistically significant associations of measures of physical fitness with METs in any other tasks indicating that absolute PA intensity increases at similar manner in lower and higher fit children. We also found that while higher fit children operated at lower level relative to their cardiovascular and metabolic capacity in several activities commonly used in accelerometry cut-off calibration studies, METs were not able to differentiate these differences between children. Therefore, our results together with others^17,18,20^, suggest that absolute MAD and MET values may underestimate PA in lower fit children because reaching absolute cut-offs requires more effort from them than from higher fit children.

MC was inversely associated with V̇O_2_ at VT during walking and running tasks, but similar associations were not found when PA intensity was assessed by MAD and METs. In contrast, children with better MC achieved higher MAD values during running on a treadmill for 8 km/h, playing hopscotch, and self-paced running than those with lower MC although MC was inversely associated with V̇O_2_ relative to V̇O_2_ at VT. These results suggest that children with higher MC can operate lower relative intensity level at a certain task than those with lower MC. In addition, accelerometry may underestimate PA intensity in children with lower MC especially in tasks considered vigorous. Finally, we found negative association patterns between MC and PA intensity defined by proportion of EMG normalised for self-paced walking at lower gait speeds suggesting that relative muscle activity is lower in children with better MC than their less competent peers. Similar association pattern was found between MC and MAD. Because we observed that MC was not associated with V̇O_2peak_ or V̇O_2_ at VT, the lower PA intensity at a certain task in children with better MC compared to those with lower MC could not be solely explained by their fitness level. Therefore, more efficient movement patterns and step frequency in children with better MC may explain these findings.

Partly in contrast with previous studies in adults^17,20^, we found few and weak associations between adiposity and the measures PA intensity in different physical activities. Nevertheless, children with higher BF% operated at higher intensity level assessed by V̇O_2_ as % of V̇O_2reserve_, V̇O_2_ as % of V̇O_2_, and MAD during climbing up and down the stairs. One reason for these partly contrasting findings may be that our sample was relatively lean. It is possible that inert load caused by excess fat mass would have caused different responses in a sample with more overweight and obese children. Furthermore, climbing up and down the stairs may be demanding activity for those with higher adiposity because the need to carry inert fat mass. However, these findings need to be confirmed in other populations.

Most observational studies using accelerometry providing evidence on the associations of PA with CRF, MC, muscle strength, and adiposity have used absolute intensity cut-offs and therefore estimated an ability of an individual to operate at certain absolute intensity level^14^. Our results suggest that absolute MAD and MET are not able to differentiate true physiological strain, which could cause remarkable misclassification of PA intensity. Therefore, the use of absolute PA intensity cut-offs in previous studies showing positive associations between PA, CRF, and MC may have clouded our understanding on the role of CRF and MC in PA and vice versa. Therefore, it seems that these previous results are partly due to the fact that higher fit and motorically more competent children reach absolute cut-offs more easily than other children. These results are supported by few studies in adults^17,18^.

The strengths of the present study include a valid and simultaneous assessment of V̇O_2_, accelerometry, and EMG during physical activities. We also assessed of V̇O_2peak_ and VT, muscle strength, MC, and adiposity using valid methodology. However, V̇O_2peak_ and VT were assessed during a maximal cycle ergometer test and V̇O_2peak_ was adjusted using the data from treadmill running or self-paced running if higher V̇O_2_ was observed during those tasks. Therefore, it is possible that we have underestimated true V̇O_2max_ in some participants and this may have had a minor effect on V̇O_2reserve_ estimation. We also determined VT in the maximal cardiopulmonary exercise test on a cycle ergometer. VT assessed during cycling may not be directly comparable that of measured during running, but the evidence is still equivocal^37^. Furthermore, relatively small sample size precluded further analyses to investigate whether the observed associations would be similar in different age or maturation groups. However, because it is common in large scales studies to utilise a single absolute acceleremetry cut-off in a sample of children and adolescents with large age-range^38–42^, our results can be used to inform further analyses and interpretation of the results of such studies. Finally, the relatively large number of analyses increases the possibility of false positive findings.

In conclusions, we found that MADs and METs as measures of PA intensity underestimated PA intensity in children with lower V̇O_2peak_, V̇O_2_ at VT, and MC. More research is warranted to develop better methods to assess PA and to take into account these individual characteristics.

## Data Availability

The datasets generated during and/or analysed during the current study are available from the corresponding author on reasonable request.

## Abbreviations

BF%: Body fat percentage
BMI: Body mass index
BMI-SDS: Body mass index standard deviation score
CHIPASE: Children’s physical activity spectrum study
CRF: Cardiorespiratory fitness
EMG: Electromyography
MC: Motor competence
MAD: Mean amplitude deviation
MET: Metabolic equivalent of task
PA: Physical activity
V̇CO_2_: Carbon dioxide production
V̇O_2_: Oxygen uptake
SMM: Skeletal muscle mass
V̇_E_: Minute ventilation
V̇O_2peak_: Peak oxygen uptake
VPA: Vigorous intensity physical activity
VT: Ventilatory threshold
